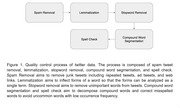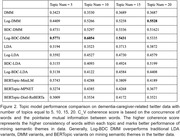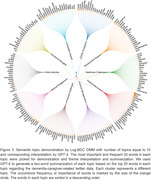# Exploring Semantic Topics in Dementia Caregiver Tweets

**DOI:** 10.1002/alz.093035

**Published:** 2025-01-09

**Authors:** Yanbo Feng, Bojian Hou, Ari Klein, Karen O'Connor, Jiong Chen, Andrés Mondragón, Shu Yang, Graciela Gonzalez‐Hernandez, Li Shen

**Affiliations:** ^1^ University of Pennsylvania, Philadelphia, PA USA; ^2^ Cedars Sinai Medical Center, West Hollywood, CA USA

## Abstract

**Background:**

Caring for family caregivers of dementia patients has grown to an important topic. Social media platforms, like Twitter, provide great resources for studying the needs of caregivers. It would be beneficial to understand the caregivers’ interested or concerned topics from their tweets. Meanwhile, topic modeling has become an efficient NLP tool for analyzing semantic themes within texts. Thus, in this study, we perform a novel topic model leveraging weighting strategies to analyze dementia‐caregiver‐related twitter data.

**Method:**

The information about the twitter data collection is available at: https://aging.jmir.org/2022/3/e39547. In our analysis, we first performed quality control, as shown in Figure 1. Then, we deployed two word‐frequency weighting strategies, *Log weighting* and *Balanced Distributional Concentration (BDC) weighting*, on the Dirichlet Multinomial Mixture (DMM) model to obtain 4 DMM variants and used them to mine semantic topics within the tweets. We compared the performance of DMM variants, LDA variants, and BERTopic variants. Topic numbers of 5, 10, 15, 20 were picked to achieve comprehensive comparison. The coherence score C_V was applied to evaluate the model performance. We then leveraged the GPT‐4 to summarize the bag of words in each topic from the best model to generate interpretable themes.

**Result:**

224,862 tweets passed the quality control and were analyzed in our study. As shown in Figure 2, the performance evaluation demonstrated that the Log‐BDC DMM has the best general performance among traditional and BERT‐based models, with an averaged coherence score of 0.5648. The bags of words from the Log‐BDC DMM with 10 topics were interpreted using GPT‐4 due to the highest coherence score of 0.6054. The interpretation yielded 10 semantic themes, as shown in Figure 3.

**Conclusion:**

Our investigation of the twitter data shows that the DMM model with Log and BDC strategies has promising power for mining meaningful semantic topics from short and noisy tweets. The model outperforms LDA models and the BERTopic models while maintaining high model interpretability. This initial study on twitter data provides interesting findings that can provide better care to family caregivers and demonstrates the promise and interpretability of traditional topic modeling methods.